# Photocatalytic performance of CuI/Cu^2+^ -TiO_2_ for controlling *Magnaporthe oryzae* infection in rice plants

**DOI:** 10.1371/journal.pone.0354519

**Published:** 2026-08-06

**Authors:** Hanh Thi Truong, Binh Quang Do, Tho Truong Pham, Ha Tien Le

**Affiliations:** 1 Optical Materials Research Group, Science and Technology Advanced Institute, Van Lang University, Ho Chi Minh City, Vietnam; 2 Faculty of Applied Technology, Van Lang School of Technology, Van Lang University, Ho Chi Minh City, Vietnam; 3 Faculty of Natural Science Education, Saigon University, Ho Chi Minh City, Vietnam; 4 Laboratory of Magnetism and Magnetic Materials, Science and Technology Advanced Institute, Van Lang University, Ho Chi Minh City, Vietnam; 5 Institute of Sciences and Technology, TNU-University of Sciences, Thai Nguyen, Vietnam; University of Salento, ITALY

## Abstract

This study presents the synthesis, characterisation, and photocatalytic antifungal performance of a Cu^+^/Cu^2+^–TiO_2_ nanocomposite designed to control rice blast disease caused by *Magnaporthe oryzae*. The heterostructure was fabricated through a chemical precipitation process in which TiO_2_ was first doped with Cu^2+^ ions, followed by partial reduction to Cu^+^ to form CuI. X-ray Photoelectron Spectroscopy (XPS) confirmed the coexistence of both Cu^+^ and Cu^2+^ oxidation states. A notable feature of this material is its Cu^+^/Cu^2+^ redox cycling, which enhances charge separation during photocatalysis and supports the sustained production of reactive oxygen species (ROS), such as ^●^O_2_^−^ and ^●^OH. These reactive species are **intensified under visible-light** LED **irradiation,** thereby imposing strong oxidative stress on the fungal pathogen. The optical bandgap energy (Eg) was calculated from UV–Vis diffuse reflectance spectra (DRS) using a Tauc plot method implemented in a Python-based analysis. Eg decreased systematically from 3.02 eV for pristine TiO_2_ to 2.67 eV for the optimized 3CuT3 composite. Correspondingly, the UV–Vis absorption edge exhibited a red shift from approximately 410–465 nm, indicating enhanced visible-light absorption. The 3CuT3 sample, containing the highest Cu loading (9.52 wt%), exhibited the strongest antifungal activity, achieving 91.55% inhibition of *M. oryzae* at 500 ppm after 10 days. In comparison, the 1CuT1 and 2CuT2 samples showed lower inhibition efficiencies of 78.22% and 83.55%, corresponding to their lower Cu contents of 2.73 wt% and 4.04 wt%, respectively. These findings suggest that the Cu species (Cu^+^/Cu^2+^) incorporated into the nanocomposite play a critical role in enhancing antifungal performance. This work provides a basis for further evaluation of the practical applicability and ecological safety of the Cu^+^/Cu^2+^–TiO_2_ nanocomposite for sustainable crop protection. ppm after 10 days. In comparison, the 1CuT1 and 2CuT2 samples showed lower inhibition efficiencies of 78.22% and 83.55%, corresponding to their lower Cu contents of 2.73 wt% and 4.04 wt%, respectively. These findings suggest that the Cu species (Cu^+^/Cu^2+^) incorporated into the nanocomposite play a critical role in enhancing antifungal performance. This work provides a basis for further evaluation of the practical applicability and ecological safety of the Cu^+^/Cu^2+^–TiO_2_ nanocomposite for sustainable crop protection.

## 1. Introduction

Rice serves as the primary food source for over half of the world’s people. Among the leading global exporters, Vietnam plays a crucial role in supplying rice to international markets, with export projections for 2025 expected to exceed 7.5 million tonnes [1]. Rice continues to be a vital crop in Vietnam, playing a crucial role in both the economy and society. Although rice farming has seen notable improvements, the crop is still highly susceptible to damaging diseases—particularly rice blast, which is caused by the fungus *Magnaporthe oryzae*, a pathogen that thrives in tropical climates. This disease presents a significant risk to global food security, potentially causing yield losses of up to 50%, severely undermining crop productivity and threatening the stability of food supplies worldwide [2,3]. In terms of economic and agronomic impact, sheath blight caused by *Rhizoctonia solani* is considered the second most damaging rice disease [4]. Furthermore, a range of other pathogenic organisms, such as *Helminthosporium oryzae*, *Gerlachia oryzae*, *Pyricularia oryzae*, *Xanthomonas oryzae*, and *Sclerotium oryzae,* have been reported across various rice-growing regions. These pathogens affect multiple plant parts, including leaves, stems, roots, and sheaths, ultimately leading to significant yield reductions and contributing to economic hardship and poverty in affected communities [5].

The widespread reliance on synthetic chemical pesticides and fungicides has raised serious concerns regarding environmental and public health. Persistent use of these chemicals contributes to biodiversity loss, contamination of water and food systems, and increased risks of chronic illnesses, including cancer and neurological disorders. The environmental accumulation of hazardous compounds such as DDT, Carbofuran, Atrazine, and Glyphosate underscores the urgent need for more sustainable and eco-friendly pest management strategies [6].

Biopesticides, including phytopesticides, microbial pesticides, and nanobiopesticides, have emerged as sustainable alternatives to conventional chemical pesticides. They provide effective pest control with lower environmental impact and reduced risk of bioaccumulation [7].

Recently, semiconductor-based photocatalysts such as TiO_2_, WO_3_, BiVO_4_, Fe_2_O_3_, and g-C_3_N_4_ have attracted attention for their antibacterial and antifungal activities. Despite their stability, low cost, and environmental compatibility, their practical application is often limited by their dependence on UV-light activation. To overcome this limitation, researchers have explored metal and non-metal doping, as well as nanocomposite approaches, to enhance photocatalytic activity under visible light. Once activated, these modified materials generate reactive oxygen species (ROS), which can disrupt microbial cell structures and inhibit microbial growth [8–13]. Among various materials, titanium dioxide (TiO_2_), a widely studied n-type semiconductor, stands out for its photostability. Metal-doped TiO_2_ has been successfully employed for the degradation of organic pollutants under visible light irradiation [14]. Metal oxide materials, including ZnO-doped TiO_2_, have shown significant antimicrobial effectiveness in inhibiting the growth of *Salmonella typhi* and *Staphylococcus aureus*. Additionally, non-metal co-doping of TiO_2_ nanoparticles (e.g., with nitrogen and fluorine) has demonstrated strong antifungal activity against fungi such as *Fusarium oxysporum.* Their colloidal nature enhances interactions with fungal cell walls, enabling complete inhibition of tomato fruits and demonstrating strong potential for solar-driven disinfection [15]. Recently, several studies have demonstrated the potential of nanomaterials for controlling plant diseases, particularly rice blast disease [16,17]. Under greenhouse conditions, nanomaterial-based treatments have been reported to reduce blast severity by up to 70% [17]. In particular, TiO₂ combined with copper or its compounds offers a dual function: acting as an antifungal agent and providing nutrients to plants—an enhancement over conventional materials such as Bordeaux mixture, a fungicide developed by a French scientist [18]. Copper-based nanomaterials exhibit antifungal activity through multiple mechanisms. Released Cu^+^/Cu^2+^ ions can induce excessive reactive oxygen species (ROS) generation, leading to oxidative stress, lipid peroxidation, protein inactivation, and DNA damage. Furthermore, copper nanoparticles can directly interact with fungal cell walls and membranes, increasing membrane permeability and causing leakage of intracellular constituents [19,20]. Elevated intracellular copper levels further disrupt metal ion homeostasis and essential metabolic pathways, ultimately inhibiting spore germination and mycelial growth [21].

Previous studies have mainly focused on Cu^2+^ doped TiO_2_, where Cu^2+^ primarily acts as an electron-trapping center to improve charge separation. However, excessive Cu^2+^ loading may also lead to charge accumulation and the formation of recombination centers, thereby limiting charge-transfer efficiency. The research gap lies in the limited exploration of mixed-valence Cu^+^/Cu^2+^ systems, in which the reversible Cu^+^/Cu^2+^ redox cycle can function as an efficient electron-shuttling pathway, facilitating the separation of photogenerated electron–hole pairs and enhancing the generation of reactive oxygen species (ROS). Recent studies have demonstrated that the valence state of copper plays a crucial role in governing charge-transfer pathways and photocatalytic performance [22,23]. Nevertheless, the specific role of Cu^+^/Cu^2+^ redox cycling in ternary CuI/Cu^2+^-TiO_2_ heterostructures and its contribution to charge separation, ROS generation, and antifungal activity remain insufficiently investigated.

Building on this approach, the present study used Cu^2+^-doped TiO_2_ as a substrate for incorporating CuI, forming a CuI/Cu^2+^-TiO_2_ heterostructure. The composite was characterised using EDX, FE-SEM, TEM, XRD, and UV-Vis DRS analyses. Its photocatalytic activity was evaluated through the degradation of *Magnaporthe oryzae*, the fungus responsible for rice blast disease, demonstrating its potential for agricultural pathogen control. A key factor influencing photocatalytic performance is the band gap energy, which was determined using advanced Python-based software.

## 2. Materials and methods

### 2.1. Materials

- Titanium dioxide (TiO_2_) powder, CAS No. 13463-67-7, purity ≥ 98% (Xilong, China)
- Copper(II) sulfate pentahydrate (CuSO₄·5H_2_O), GRM677-500G, purity ≥ 99.5% (HiMedia, India)
- Potassium iodide (KI), purity ≥ 99% (HiMedia, India)
- Sodium borohydride (NaBH_4_), GRM10345-100G, purity ≥ 98% (HiMedia, India)

### 2.2. Methods

#### 2.2.1. Synthesis of CuI/Cu^2^^+^-TiO_2_ nanocomposite.

**• Step 1: Preparation of Cu^2^**^+^–**doped TiO_2_ (Cu^2^**^+^ –**TiO**_**2**_**).**

TiO₂ powder (50 g) was dispersed in 50 mL of Cu^2+^ ion solutions with concentrations of 0.02, 0.04, and 0.06 mol, respectively. The mixtures were magnetically stirred for uniform adsorption of Cu^2+^ onto the TiO_2_ surface. The resulting suspensions were then dried at 70 °C and subsequently calcined at 550 °C for 3 h to obtain Cu^2+^–doped TiO_2_, denoted as **1CuT**, **2CuT**, and **3CuT**, respectively.

- **Step 2: Synthesis of CuI/Cu^2^**^+^–**TiO_2_ nanocomposites**

CuI was prepared via a chemical precipitation route. Cu^2+^ precursor solutions (0.01, 0.02, and 0.03 mol in 35 mL solvent) were stirred at 60 °C for 30 min, followed by the dropwise addition of 10 mL of KI solution at a 1:1 molar ratio with Cu^2+^. Subsequently, 5 mL of NaBH_4_ solution, having the same molar concentration as Cu^2+^ and I^−^, was added to reduce Cu^2+^ to Cu^+^, thereby facilitating the formation of CuI under continuous stirring for an additional 1 h.

The freshly formed CuI precipitate was immediately combined with the previously prepared Cu^2+^–TiO_2_ powders (25 g each of 1CuT, 2CuT, and 3CuT), under continuous stirring to ensure uniform deposition of CuI onto the Cu^2+^ –TiO_2_ surface The CuI/Cu^2+^–TiO_2_ composite was collected by ultracentrifugation (Ultra 5.0, Hanil, Korea) at 30,000 rpm for 20 min, followed by washing and redispersion in 50 mL of absolute ethanol. This washing process was repeated three times to remove residual impurities. Finally, the products were dried at 60 °C to obtain the CuI/Cu^2+^–TiO_2_ nanocomposites. The obtained nanocomposites were designated as 1CuT1, 2CuT2, and 3CuT3, corresponding to CuI loadings derived from 0.01, 0.02, and 0.03 mol of Cu^2+^ precursor, respectively.

#### 2.2.2. Physicochemical properties of the CuI/Cu^2^^+^–TiO₂ nanocomposite.

The elemental composition and spatial distribution of the samples were analysed by energy-dispersive X-ray spectroscopy coupled with elemental mapping, carried out on a scanning electron microscopy platform (JEOL JSM-IT200). High-resolution transmission electron microscopy was employed to examine the nanoparticle morphology and lattice features, using a JEM-2100 instrument operated at an accelerating voltage of 120 kV. Particle size and size distribution were determined from micrographs using ImageJ software, and the resulting data were further processed in Microsoft Excel.

Surface morphology was investigated by field-emission scanning electron microscopy under an accelerating voltage of 10 kV and a beam current of 30 mA, with all samples sputter-coated with a conductive platinum layer prior to observation. The optical characteristics of the CuI/Cu^2^ ⁺ –TiO_2_ nanocomposite were evaluated through UV–visible diffuse reflectance spectroscopy using a JASCO V-770 spectrophotometer. BaSO_4_ was employed as the reference standard. The spectra were collected over the wavelength range of 200–800 nm with a scanning interval of 1 nm, a scan rate of 400 nm min^−1^, and a single scan (n = 1). The response time was set to 0.24 s, and the spectral bandwidth was 5.0 nm. Crystalline phase identification was conducted by X-ray diffraction with CuKα radiation (λ = 1.5405 Å) on a Bruker D8 Advance diffractometer. The measurements were carried out over a full scanning range of 10–80° (2θ). Data were collected with a step size of 0.02° (2θ) and a scan speed of 2**°** min^−1^. For clarity, only the diffraction patterns in the 20–80° range are presented in the manuscript. X-ray photoelectron spectroscopy (XPS) analysis was performed using a Thermo Scientific Nexsa G2 XPS system equipped with an Al Kα X-ray source.

- Method for estimating bandgap energy:

The Python script calculates the bandgap energy (Eg) using the Tauc plot method. First, the UV–Vis data (wavelength and absorbance) is imported and converted into photon energy (hν) and the absorption coefficient (α). Next, the Tauc variable (αhν)^1/n^ is computed, where the exponent *n* must be set to 2 for a direct bandgap or 1/2 for an indirect bandgap. A suitable linear region of the Tauc plot is then selected, and linear regression is applied to this segment. Finally, Eg is obtained by extrapolating the fitted line to the photon-energy axis (i.e., the point where the Tauc variable becomes zero), following the relation: **Eg** = **– intercept/slope.**

#### 2.2.3. Antifungal assay of CuI/Cu^2^^+^–TiO_2_ colloidal against *Magnaporthe oryzae.*

The antifungal experiments against *Magnaporthe oryzae* were conducted entirely under controlled *in vitro* laboratory conditions and did not involve field trials. Therefore, no specific permits or field-sampling authorizations were required.

The fungal strain *Magnaporthe oryzae (M. oryzae)* was obtained from the culture collection of the Microbial Technology Laboratory, Ho Chi Minh City Biotechnology Center. The isolate had been previously collected from rice leaves infected with blast disease and deposited in the culture collection. Following identification, the strain was preserved in 25% glycerol at −80 °C and routinely monitored for viability at six-month intervals.

**Fresh cultures of** *M. oryzae* **were grown on** potato dextrose agar (PDA**) for experimental purposes. The** PDA **medium was prepared by dissolving 200 g/L potato extract, 20 g/L glucose, and 20 g/L agar in 1000 mL of distilled water**, followed by autoclaving at 121 °C for 15 min. Colloidal CuI/Cu^2+^–TiO_2_ composites were added to the medium after cooling to 50 °C at concentrations of 0, 20, 40, 60, 80, 100, 300, and 500 ppm. The mixture was thoroughly homogenized, poured into sterile Petri dishes (90 × 15 mm), and allowed to solidify. Each Petri dish was centrally inoculated with a 6 mm diameter mycelial disc, excised from the actively growing margin of a *Magnaporthe oryzae* colony on PDA. The plates were then exposed to visible light using a white LED source (400–700 nm) with an intensity of approximately 20–25 mW.cm^−2^, positioned 10 cm above the samples, for 30 min.

The plates were subsequently incubated at ambient temperature, and radial mycelial growth was measured daily for up to 10 days. The extent of radial growth inhibition was assessed once the fungal growth in the control plates reached the dish's edge. The percentage of mycelial growth inhibition (E%) was calculated according to the following equation [24]:


$$\begin{array}{c} E\left(\;\%\right)=\frac{\left(dc-dt\right)}{dc}\times 100\; \end{array}$$ (1)

Where dc represents the diameter of the mycelial growth in the untreated control grown on PDA medium only, and dt denotes the diameter of the mycelial growth in the sample treated with CuI/Cu^2+^–TiO_2_ following the incubation period.

#### 2.2.4. Statistical analysis.

All experiments were performed in triplicate, and the data are reported as mean values with their corresponding standard deviations (±SD). For the antifungal study, a two-factor ANOVA was employed to assess the effects of concentration and band gap energy on fungal growth inhibition by the 1CuT1, 2CuT2, and 3CuT3 nanocomposites relative to the control treatment. When significant differences were detected, Fisher’s Least Significant Difference (LSD) post-hoc test was applied for pairwise comparisons at a 95% confidence level (α = 0.05), following established statistical procedures [25].

The critical threshold for pairwise comparisons was calculated using Fisher’s LSD according to Equation (2):


$$\begin{array}{c} LSD=t_{crit}\;\times \sqrt{\frac{\text{2}\times {MS}_{Error}}{n}} \end{array}$$ (2)

Where MSError is the mean square error obtained from the ANOVA, n is the number of replicates per group, and t_crit_ is the critical t-value at the selected significance level.

## 3. Results and discussion

### 3.1. Fabrication CuI/Cu^2+^–TiO₂ nanocomposite

The CuI/Cu^2+–^TiO_2_ **nanocomposite** was synthesised through a **precipitation reaction** between Cu^2+^ and I^−^ ions in solution using NaBH_4_ as a reducing agent, followed by **calcination** of the resulting Cu^2+^–TiO_2_ precursor. The reaction outlined below depicts the overall synthesis process:


$$\begin{array}{c} {\text{Cu}}^{\text{2}+}\;+\;{\text{I}}^{-}\;+\;{\text{BH}}_{\text{4}}^{-}\;+\;{\text{3H}}_{\text{2}\;}\text{O }\to \text{ CuI}↓\;+\;{\text{B}(\text{OH})}_{\text{3}}\;+\;{\frac{\text{7}}{\text{2}}\text{H}}_{\text{2}\;}↑ \end{array}$$ (3)

**Fig 1:**
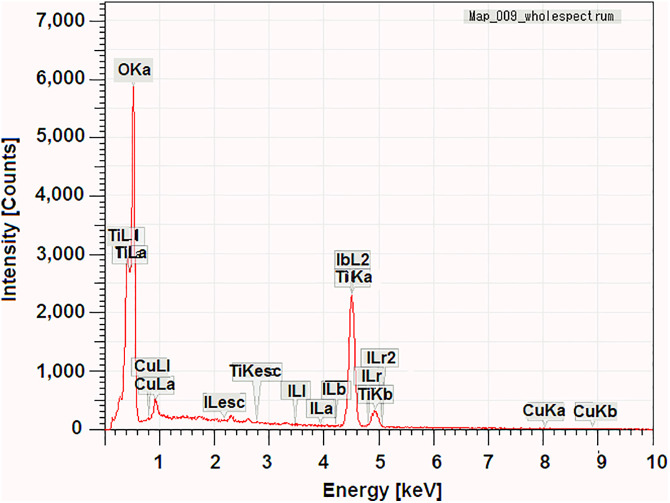
EDS spectrum of the CuI/Cu^2+^–TiO_2_ nanocomposite.

The formation and composition of the CuI/Cu^2^ ⁺ –TiO₂ nanocomposites were confirmed by energy-dispersive X-ray spectroscopy (EDS), as shown in Fig 1. The EDS spectrum revealed characteristic peaks corresponding to **Ti** (0.5 keV and 4–5 keV), **Cu** (1 keV and 8–9 keV), **I** (2–5 keV), and **O** (a strong peak near 0.5 keV). These results are consistent with previous reports on EDS analyses of Cu- and CuO-modified TiO₂-based composites [26–28]. The CuI/Cu^2^ ⁺ –TiO₂ nanocomposite was synthesised by a precipitation reaction between Cu^2^⁺ and I⁻ ions in solution using NaBH₄ as a reducing agent, followed by mixing with the pre-calcined Cu^2^ ⁺ –TiO_2_ precursor.

**Fig 2:**
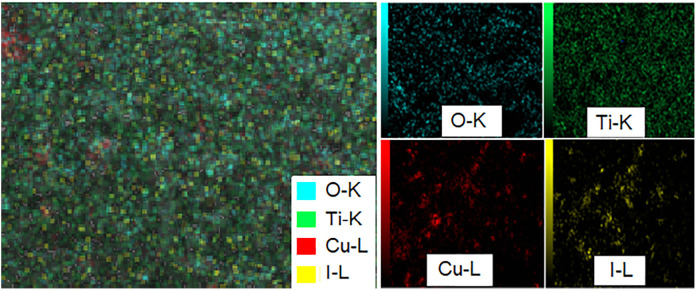
Elemental mapping images of the CuI/Cu² ⁺ –TiO₂ nanocomposite.

Elemental mapping images (Fig 2) further confirm the homogeneous distribution of Cu, I, Ti, and O throughout the nanocomposites. As summarised in Table 1, the Cu and I contents increased proportionally with the concentrations of Cu^2+^ and I^−^ precursors, supporting the successful incorporation of CuI within the Cu^2+^–TiO₂ matrix. Specifically, the Cu mass percentage increased from 2.73% to 4.04% and 9.52% in 1CuT1, 2CuT2, and 3CuT3, respectively, corresponding to total Cu^2+^ inputs of 0.02, 0.04, and 0.06 mol per 25 g of nanocomposite over the two synthesis steps. Similarly, the I content rose from 0.64% to 1.94% and 8.03%, consistent with KI precursor levels of 0.01, 0.02, and 0.03 mol per 25 g. Overall, the EDS and elemental mapping results validate the successful fabrication of the CuI/Cu^2+^–TiO₂ nanocomposites, providing a stable structural foundation for their subsequent photocatalytic performance.

**Table 1:** Mass and atomic compositions of 1CuT1, 2CuT2, and 3CuT3 at Cu^2+^ concentrations of 0.01, 0.02, and 0.03 mol, respectively.

Element	1CuT1		2CuT2		3CuT3	
	Mass%	Atom%	Mass%	Atom%	Mass%	Atom%
**O**	37.72 ± 0.21	64.85 ± 0.36	36.66 ± 0.21	64.23 ± 0.36	31.19 ± 0.18	60.30 ± 0.35
**Ti**	58.91 ± 0.47	33.83 ± 0.27	57.36 ± 0.47	33.56 ± 0.28	51.26 ± 0.45	33.11 ± 0.29
**Cu**	2.73 ± 0.09	1.18 ± 0.04	4.04 ± 0.11	1.78 ± 0.05	9.52 ± 0.15	4.63 ± 0.07
**I**	0.64 ± 0.13	0.14 ± 0.03	1.94 ± 0.16	0.43 ± 0.04	8.03 ± 0.25	1.96 ± 0.06
**Total**	100.00	100.00	100.00	100.00	100.00	100.00

### 3.2. Characterisation of CuI/Cu^2+^–TiO_2_ nanocomposite

**Fig 3:**
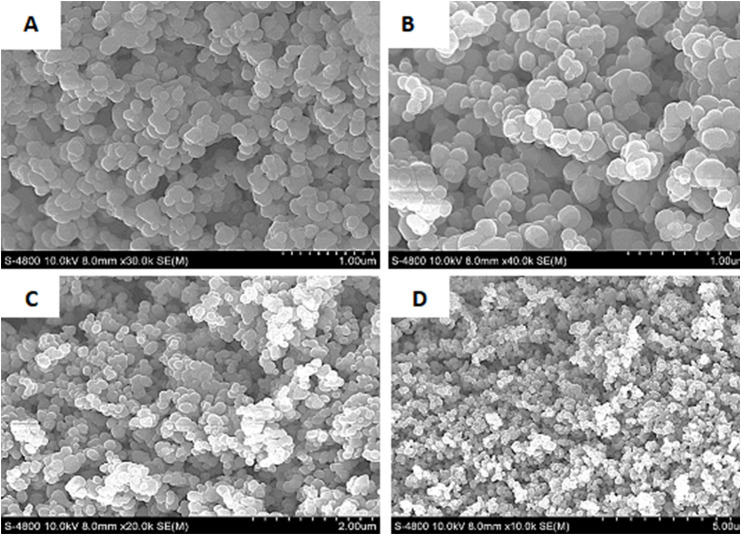
FE-SEM micrographs of Cu^2+^–TiO_2_ (A) and CuI Cu^2+^−TiO_2_ with scale bars of 1 μm (B), 2 μm (C), and 5 μm (D).

Fig 3 shows the FE-SEM images of calcined Cu^2+^-TiO₂ and the CuI/Cu^2+^–TiO₂ nanocomposite. The surface of Cu^2+^-TiO₂ appears relatively smooth (Fig 3A), whereas the nanocomposite exhibits a thicker texture with distinguishable light and dark contrast, indicating the coexistence of two phases (Fig 3C-3D). This contrast is attributed to the distribution of CuI nanoparticles deposited on the Cu^2+^–TiO₂ surface. In these images, the CuI domains appear as the lighter (brighter) phase, while TiO₂ appears darker. These observations are consistent with previous reports on binary nanocomposites, in which secondary nanoparticle deposition increases surface roughness and produces heterogeneous morphology, resulting in differential electron scattering [11,29,30].

**Fig 4:**
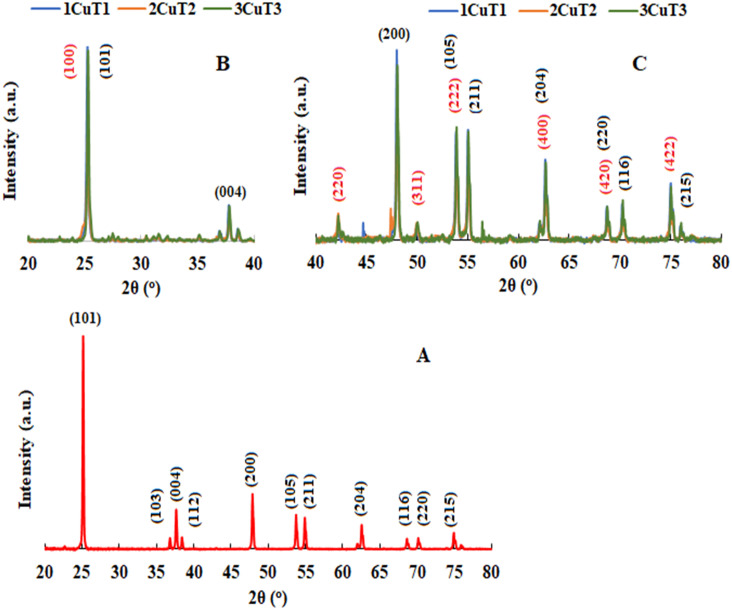
XRD patterns of TiO_2_ (A) and the CuI/Cu^2+^–TiO_2_ nanocomposite (B, C).

**The** XRD **pattern of Cu^2^**^+^**-TiO₂ calcined at 550 °C for 3 hours is shown** in Fig 4A.The strongest diffraction signal observed near 25.21° is attributed to the (101) lattice plane of anatase-phase TiO₂. **Other diffraction peaks located at 2**θ = **37.69°, 47.93°, 53.82°, 54.96°, 62.59°, 68.67°, 70.16°, and 74.96° are assigned to the (004), (200), (105), (211), (204), (116), (220), and (215) planes, respectively. These results are in good agreement with the standard diffraction data for anatase TiO₂ (**JCPDS **00-021-1272) [**8,14,31**].**

The XRD profile of the CuI/Cu^2+^–TiO₂ heterostructure, shown in Fig 4B (20–40°) and Fig 4C (40–80°), exhibits diffraction peaks positioned similarly to those of Cu^2+^–TiO_2_. Upon the deposition of CuI onto Cu^2+^–TiO_2_, the main peaks of γ-CuI overlap with the characteristic reflections of anatase TiO_2_. Specifically, the diffraction peaks observed at 2θ = 25.24°, 53.94°, 62.70°, 68.81°, and 75.08° correspond to the (100), (222), (400), (420), and (422) crystal planes of γ-CuI, respectively, and are highlighted in red in Fig 4B and 4C. Furthermore, the emergence of new diffraction peaks at 42.21° and 50.07°, indexed to the (220) and (311) planes of γ-CuI, aligns well with the standard reference pattern (JCPDS 06–0246) [32,33]. The appearance of these additional peaks confirms the coexistence of two crystalline phases within the CuI/Cu^2+^–TiO₂ nanocomposite.

**Fig 5:**
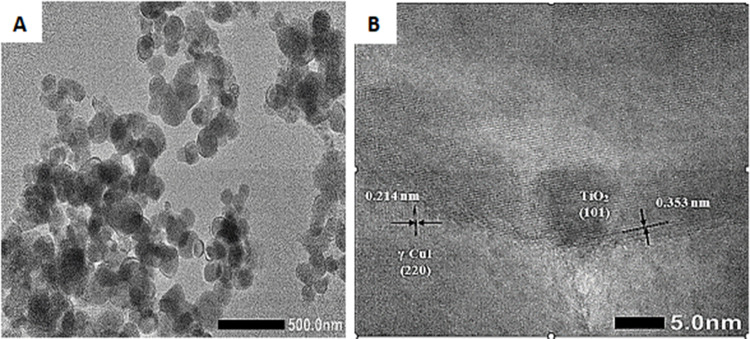
TEM (A) and HRTEM (B) micrographs of the CuI/Cu–TiO_2_ nanocomposite (2CuT2).

The TEM micrograph in Fig 5A shows that the CuI/Cu^2+^–TiO_2_ nanocomposite exhibits nearly spherical nanoparticles distributed on the surface. The corresponding HRTEM image in Fig 5B displays well-defined lattice fringes with an interplanar spacing of 0.353 nm, corresponding to the (101) planes of anatase TiO₂. In addition, a lattice spacing of 0.214 nm is assigned to the (220) planes of γ-CuI. These findings further confirm the coexistence of two crystalline components within the synthesised nanocomposite. Similar determinations of lattice spacings in heterogeneous TiO_2_-based nanostructures have also been reported by other researchers [9,34].

**Fig 6:**
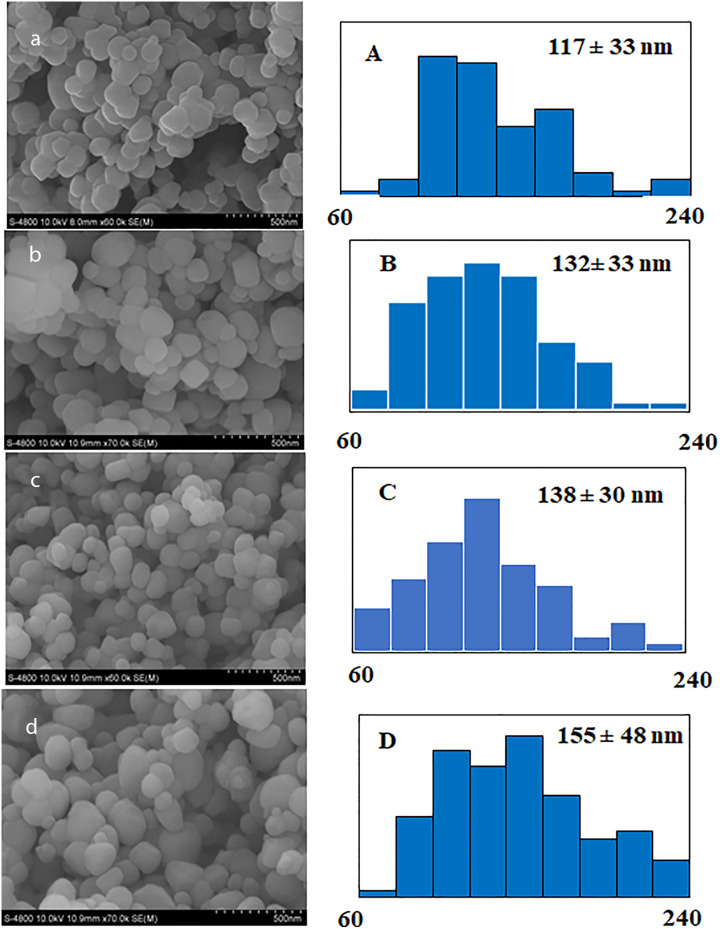
FE-SEM images of (a) TiO_2_, (b) 1CuT1, (c) 2CuT2, (d) 3CuT3, and (A–D) their corresponding particle size distribution histograms (plotted with a uniform bin width of 20 nm).

The average particle size of the CuI/Cu^2+^–TiO_2_ nanocomposites was determined from FE-SEM micrographs by measuring 100 randomly selected particles using ImageJ to obtain a representative size distribution. The calcined Cu^2+^–TiO_2_ sample exhibited a nearly spherical morphology with slight agglomeration and an average diameter of 117 ± 33 nm (Fig 6A). Following the introduction of CuI via precipitation, particle sizes increased with higher CuI loading, as summarised in Table 1. The 1CuT1 and 2CuT2 samples displayed average diameters of approximately 130–140 nm (Fig 6B and 6C), whereas the 3CuT3 sample, containing the highest CuI content, showed a larger mean size of 155 ± 48 nm (Fig 6D). Particles in the 3CuT3 sample appeared less homogeneous, exhibiting a broader size range. Nevertheless, all samples exhibited relatively narrow size-distribution histograms (Fig 6A–6D), indicating good overall uniformity in particle dimensions. This trend aligns with previous reports on nanocomposites composed of metal oxides combined with additional metal or metal-oxide phases [28,35–37].

**Fig 7:**
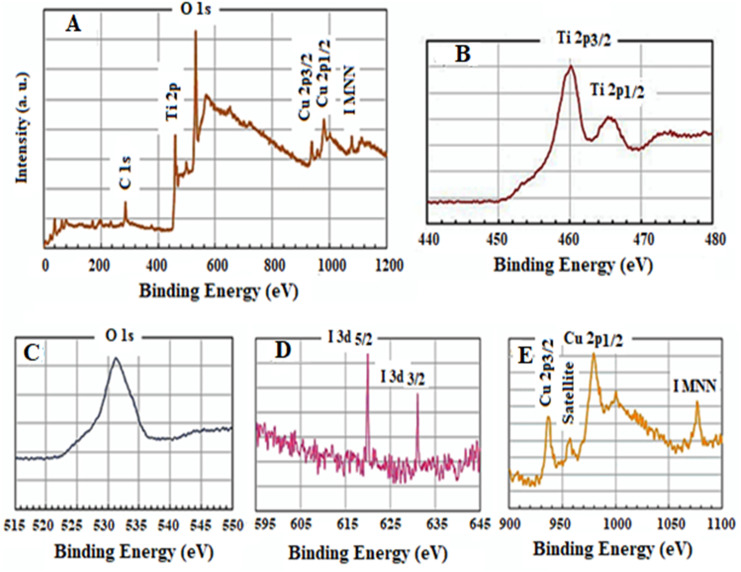
XPS spectra of the CuI/Cu^2+^–TiO_2_ nanocomposite survey spectrum (A). Elemental composite: Ti 2p (B), O 1s (C), I 3d (D), and Cu 2p with the I MNN Auger feature (E).

The X-ray photoelectron spectroscopy (XPS) analysis was performed to investigate the elemental composition, chemical states, and interfacial interactions within the CuI/Cu^2+^–TiO₂ nanocomposite. The survey spectrum confirmed the presence of Ti, Cu, I, and O, verifying the successful formation of the composite structure (Fig 7A).

The Ti 2p spectrum shows two distinct peaks at 450.5 eV (Ti 2p_3/2_) and 465.7 eV (Ti 2p_1/2_), characteristic of Ti^4+^ in the TiO_2_ lattice, indicating strong interfacial coupling between Cu-based species and TiO₂ (Fig 7B). This electronic interaction facilitates favourable band alignment and enhances charge-carrier mobility in the heterostructure. The O1s spectrum can be deconvoluted into two components: lattice oxygen (O^2^⁻) at 531.5 eV and surface oxygen attributed to hydroxyl groups or oxygen vacancies (Fig 7C). The presence of oxygen vacancies provides active sites for molecular adsorption and electron trapping, thereby improving photocatalytic performance.

In the iodine region, the I 3d peaks are clearly detectable at 619.98 eV (I 3d_5/2_) and 631.08 eV (I 3d_3/2_) (Fig 7D). A strong feature observed at ~1071 eV corresponds to the I MNN Auger trans ition, which is expected because Auger processes are dominant in heavy atoms and MNN Auger electrons exhibit high escape probability (Fig 7E). The appearance of these iodine signals is consistent with a previous report on iodide-containing nanocomposites. The incorporation of iodine into the nanocomposite promotes charge carrier mobility and induces favourable alterations in the band structure, leading to enhanced photocatalytic efficiency [38]. The carbon C1s signal at ~285.18 eV was used as the calibration standard [39].

In the Cu 2p region, two main peaks were observed at approximately 938 eV (Cu 2p_3/2_) and 980 eV (Cu 2p_1/2_), characteristic of Cu^+^ species originating from CuI. A weak satellite peak at 957 eV further indicates the presence of Cu^2+^ (Fig 7E). The coexistence of Cu^+^ and Cu^2+^ suggests partial reduction of Cu^2+^ to Cu^+^ and strong electronic interactions between CuI and TiO₂ at the heterojunction interface. The Cu^+^/Cu^2+^ redox couple is known to promote charge separation and electron transfer during photocatalysis, aligning with the authors’ approach—introducing Cu^2+^ into TiO_2_ before calcination and subsequently reducing it with NaBH_4_ to form CuI. The chemical interactions among Cu^+^, Cu^2+^, O, and Ti are consistent with previously reported findings [35,40].

Overall, the XPS results confirm the coexistence of Cu^+^ and Cu^2+^ species and reveal strong interfacial electronic interactions between CuI and TiO_2_. This synergistic effect facilitates efficient charge separation and ultimately contributes to the enhanced photocatalytic performance of the CuI/Cu^2+^–TiO_2_ nanocomposite.

### 3.3. Optical properties and antifungal activity of CuI/Cu^2+^-TiO_2_

#### 3.3.1. Optical properties.

**Fig 8:**
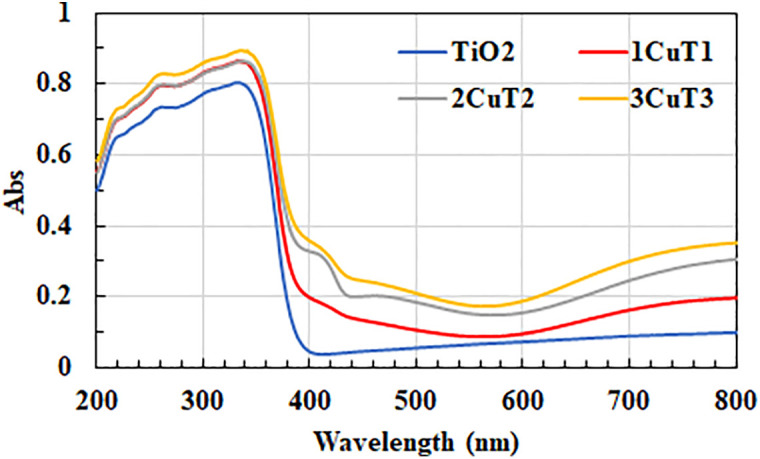
DRS UV–Vis spectra of TiO_2_ and the CuI/Cu^2+^–TiO_2_ nanocomposite.


The DRS UV–Vis spectra of TiO_2_ and the CuI/Cu^2+^–TiO_2_ nanocomposites recorded in the wavelength range of 200–800 nm (Fig 8) were used to determine the optical bandgap energies of the samples. The absorption edge exhibited a red shift from approximately 410 nm for TiO_2_ to 465 nm for the 3CuT3 nanocomposite, indicating enhanced visible-light absorption with increasing CuI loading. In this study, the bandgap values were calculated using a Python-based Tauc-plot script following the equation [8–10]:

$$\begin{array}{c} \left({\alpha hv)}^{\frac{\text{1}}{n}}=B(hv-Eg\right) \end{array}$$ (4)

**Fig 9:**
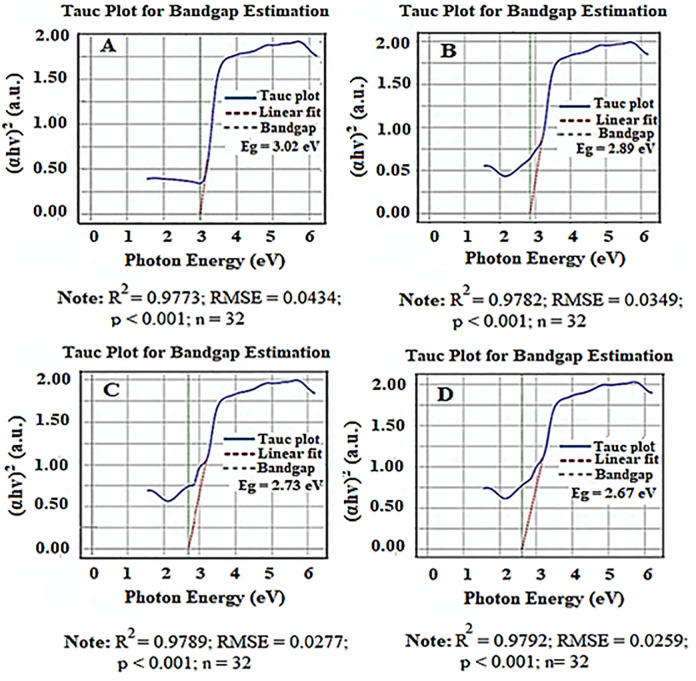
Tauc plots and the corresponding band gap energies of TiO_2_ (A), 1CuT1 (B), 2CuT2 (C), and 3CuT3 (D).

Where *n* = ½ for indirect allowed transitions typical of TiO_2_. The pristine TiO_2_ exhibited a bandgap of 3.02 eV (Fig 9A), which is consistent with the reported values for anatase-phase TiO_2_. Upon incorporation of Cu species, a gradual and systematic narrowing of the bandgap was observed with increasing Cu loading (2.73%, 4.04%, and 9.52% Cu/TiO_2_; Table 1). The estimated bandgap energies decreased from 2.89 eV for 1CuT1 to 2.73 eV for 2CuT2 and further to 2.67 eV for 3CuT3 (Fig 9B–9D), indicating that Cu incorporation effectively extended the optical absorption of TiO_2_ toward the visible-light region. The reliability of the bandgap determination was confirmed by the high quality of the fitting results, which exhibited R^2^ values close to 0.98, RMSE values of approximately 0.04, p-values below 0.001, and more than 32 fitting points for each selected region. These statistical parameters demonstrate excellent fitting performance and support the accuracy, robustness, and reproducibility of the extracted bandgap values.

This red shift in absorption edge and reduction in bandgap can be attributed to the formation of Cu^+^/Cu^2+^ energy levels within the TiO_2_ band structure and the strong interfacial interaction between CuI, Cu^2+^, and TiO_2_. The introduction of CuI contributes I-3d and Cu-2p states near the valence band, while Cu^2+^ species facilitate the formation of defect states and enhance visible-light harvesting. Similar bandgap narrowing trends have been reported for Cu-modified TiO_2_ nanocomposites, confirming that Cu doping or coupling promotes enhanced optical absorption and improved photocatalytic performance [41–44].

To elucidate the photocatalytic behaviour of the semiconductor nanocomposite, it is essential to estimate the energy positions of the conduction band (CB) and valence band (VB). These parameters can be determined using established empirical relationships [11]:


$$\begin{array}{c} {\text{E}}_{\_(\text{VB})\;}\;=\;X_{m}\;-\;{\text{E}}_{\_(\text{e})}+\;\text{1}/\text{2}{\text{E }}_{\text{g}} \end{array}$$ (5)

$$\begin{array}{c} {\text{E }\_}_{(\text{CB})\;=}\;{\text{E }\_}_{\left(\text{VB}\right)}-\;{\text{E }}_{\text{g}} \end{array}$$ (6)

In these expressions, E__(VB)_ and E__(CB)_ represent the valence and conduction band edge potentials, respectively; X_m_ denotes the absolute electronegativity of the semiconductor; E__(e)_ refers to the energy of free electrons on the hydrogen reference scale (4.5 eV); and E_g_ corresponds to the band gap energy. The value of X_m_ is obtained from the geometric mean of the electronegativities of the constituent elements, each weighted by its atomic proportion in the material [45]. The Mulliken electronegativity, in turn, is expressed as the average of the ionisation energy and electron affinity [46,47]:


$$\begin{array}{c} X_{m}=\;\frac{\text{1}}{\text{2}}\;{[\text{E}}_{\text{ionization }}\;+\;{\text{E}}_{\text{electron affinity}}] \end{array}$$ (7)

**Scheme 1:**
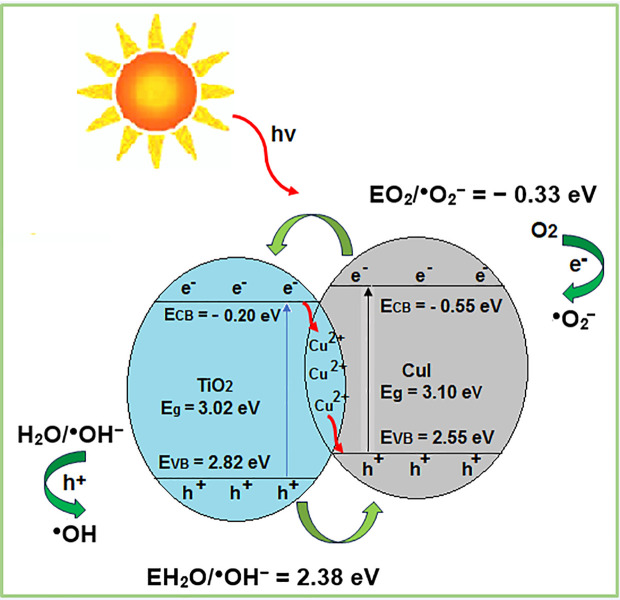
Proposed photocatalytic mechanism of the Cu^2+^-TiO_2_/CuI nanocomposite generating ^●^O₂⁻ and ^●^OH radicals.

The calculated values of X_m_, E_(_CB)_, and E__(VB)_ are summarised in Table 2, based on the experimentally determined band gap of TiO₂ (Eg = 3.02 eV) and the reported band gap of CuI (Eg = 3.10 eV) [48,49]. These results were subsequently used to construct the photocatalytic mechanism of the CuI/Cu^2+^−TiO_2_ system, as depicted in Scheme 1.

**Table 2:** The electronegativity (X) and the calculated edge positions of the CB and VB edges (E _(CB)_ and E _(VB)_) for CuI and TiO_2_.

Component	E _Ionization_ (eV)	E _Electron affinity_ (eV)	X_m_ (eV)	E__(VB)_ (eV)	E__(CB)_ (eV)
Cu	7.726	1.228	4.477		
I	10.451	3.059	6.755		
CuI			5.499	2.55	− 0.55
Ti	6.828	0.079	3.454		
O	13.618	1.461	7.540		
TiO_2_			5.812	2.82	− 0.20

When TiO_2_ is doped with Cu^2+^, the substitution of Cu^2+^ for Ti^4+^ within the TiO_2_ lattice induces charge imbalance, leading to the formation of oxygen vacancies. These defects not only enhance visible-light absorption but also facilitate charge transport within the semiconductor. Upon contact between CuI and Cu^2+^ − TiO_2_, electron redistribution occurs until Fermi-level equilibrium is established, resulting in the formation of an internal electric field and band bending at the heterojunction interface. Under light irradiation (hν), both semiconductors are photoexcited, generating electron–hole pairs.

As illustrated in Scheme 1, the photocatalytic mechanism of the CuI/Cu^2+^−TiO_2_ heterojunction is more appropriately described by a direct Z-scheme pathway rather than a conventional Type-II mechanism. In this process, the photogenerated electrons in the conduction band (CB) of TiO_2_ (ECB = −0.20 eV) migrate toward the interface and recombine with the photogenerated holes in the valence band (VB) of CuI (EVB = +2.55 eV). The Cu^2+^ species and oxygen-vacancy defects introduced into the TiO_2_ lattice serve as electron-trapping centers, facilitating interfacial charge transfer and promoting selective charge recombination. As a result, the charge carriers with the strongest redox capability are preserved. The highly reducing electrons remain in the CB of CuI (−0.55 eV), while the strongly oxidizing holes accumulate in the VB of TiO₂ (+2.82 eV). This configuration effectively maintains the superior reduction and oxidation potentials required for photocatalytic reactions.

Oxidation and reduction reactions produce radicals at the conduction and valence bands when the potentials align with those of the O_2_/^●^O₂⁻ (–0.33 eV) while H₂O/^●^OH (+2.38 eV) redox couples, respectively [50]. According to the band-edge positions listed in Table 2, the CB potential of CuI (−0.55 eV) is more negative than the O₂/•O_2_ ⁻ redox potential (−0.33 eV), enabling the reduction of dissolved oxygen to superoxide radicals according to:


$$\begin{array}{c} {\text{O}}_{\text{2}}\;+\;{\text{e}}^{-}\;\to \;\cdot {\text{O}}_{\text{2}}^{-} \end{array}$$ (8)

Meanwhile, the VB potential of TiO₂ (+2.82 eV) is more positive than the H₂O/^●^OH redox potential (+2.38 eV), allowing photogenerated holes to oxidize water or hydroxide ions into hydroxyl radicals:


$$\begin{array}{c} {\text{H}}_{\text{2}}\text{O}/{\text{OH}}^{-}\;+\;{\text{h}}^{+}\;\to \;\cdot \text{OH } \end{array}$$ (9)

Therefore, both •O_2_⁻ and •OH radicals can be efficiently generated during photocatalysis, significantly enhancing the degradation of pollutants. Furthermore, increasing the Cu^2+^ dopant concentration and CuI content promotes charge separation and accelerates reactive oxygen species (ROS) generation, thereby improving the overall photocatalytic performance of the CuI/Cu^2+^−TiO_2_ heterojunction system. These findings are consistent with the Z-scheme photocatalytic mechanism reported in recent studies [39,51,52].

#### 3.3.2. Antifungal activity of CuI/Cu^2+-^TiO_2_.

The antifungal activity of the CuI/Cu^2^ ⁺ –TiO_2_ nanocomposites against *Magnaporthe oryzae*, the causal agent of rice blast disease in tropical rice production, was evaluated using the samples 1CuT1, 2CuT2, and 3CuT3, which contain progressively higher amounts of Cu and I (from CuI) as shown in Table 1.

**Fig 10:**
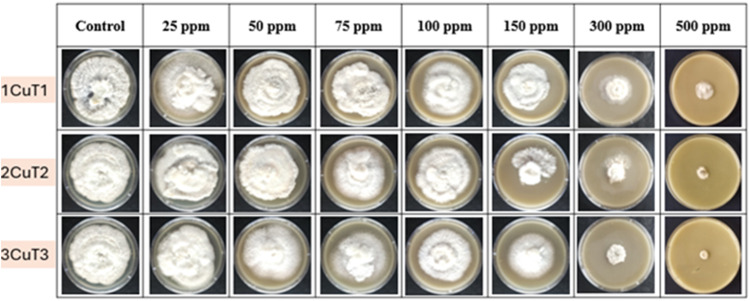
Images showing the growth inhibition of M. oryzae treated with CuI/Cu^2+^–TiO₂ nanocomposites: At concentrations of 25, 50, 75, 100, 150, 300, and 500 ppm after 240 h (10 days) of incubation.

As illustrated in Fig 10, fungal mycelia in the untreated control plates containing only PDA medium grew vigorously and reached the edge of the Petri dishes. In contrast, clear growth inhibition was observed on the plates treated with 1CuT1, 2CuT2, and 3CuT3. From left to right, as the concentration of the active material increased, the radial growth of the mycelia decreased correspondingly, with the difference becoming particularly pronounced at higher concentrations. Notably, at the highest concentration (500 ppm), the background of the culture plates turned slightly brown, and the visible mycelial diameter became extremely small.

**Fig 11:**
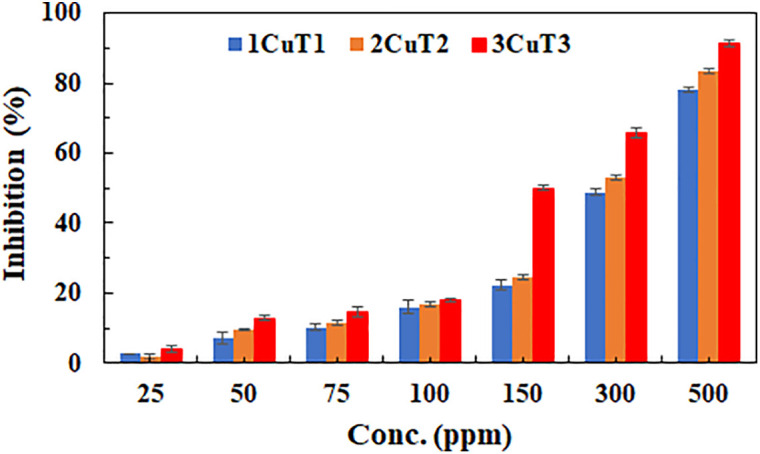
Growth inhibition of M. oryzae by CuI/Cu^2+^–TiO_2_ nanocomposites at concentrations of 25–500 ppm after 240 h (10 days) of incubation. Note: Data are expressed as mean ± SD (n = 3). Error bars represent the standard deviation of three independent experiments.

Quantitative data in Table 3 reinforce this trend, showing a consistent increase in inhibition percentage from top to bottom and left to right as the nanocomposite concentration increased. After 10 days of incubation, the fungal colony diameter in the 3CuT3 sample — containing the highest Cu loading — was reduced to only 6 mm, compared with 75 mm in the PDA control, demonstrating the strong antifungal activity and reliability of the synthesis. This trend is clearly illustrated in Fig 11, where the inhibition rate increases consistently with higher concentrations of nanocomposites containing 2.72%, 4.04%, and 9.52% Cu for the 1CuT1, 2CuT2, and 3CuT3 samples, respectively (Table 1). At 500 ppm − corresponding to 13.6, 20.2, and 47.6 ppm of total Cu^+^/Cu^2+^ in 1CuT1, 2CuT2, and 3CuT3—all samples achieved more than 78% inhibition after 10 days. Notably, the 3CuT3 sample exhibited the highest inhibition rate of ~92%. These preliminary *in vitro* results highlight the potential of the CuI/Cu^2+^–TiO₂ nanocomposite as an effective antifungal agent against *M. oryzae*. The concentration range selected in this study is consistent with those reported in previous investigations. Similar dose levels have been employed to evaluate the antifungal activity of Cu-containing materials, including CuO against *Botrytis cinerea* and *Fusarium oxysporum*, as well as Cu/TiO₂ against *Phytophthora palmivora* [53,54].

**Table 3:** Inhibition efficiency of CuI/Cu^2+^–TiO₂ nanocomposites against *Magnaporthe oryzae* after 240 h (10 days) of incubation at different concentrations.

		1CuT1		2CuT2		3CuT3	
		d (nm)	Inhibition (%)	d (mm)	Inhibition (%)	d (mm)	Inhibition (%)
	**Ctrl**	75 ± 0.3	0.00 ± 0.00^a^	75 ± 0.3	0.00 ± 0.00^a^	75 ± 0.3	0.00 ± 0.00^a^
**Concentration (ppm)**	**25**	73 ± 0.4	2.67 ± 0.00^a^	73 ± 0.4	2.00 ± 0.77^a^	71 ± 10.6	4.00 ± 0.77^a^
	**50**	70 ± 1.0	7.11 ± 1.54^a^	68 ± 0.4	9.53 ± 1.32^ab^	65 ± 0.9	12.89 ± 0.77^b^
	**75**	67 ± 1.0	10.22 ± 0.77^a^	66 ± 0.3	11.55 ± 0.77^a^	64 ± 0.7	14.66 ± 1.33^a^
	**100**	63 ± 0.9	16.00 ± 1.88^a^	62 ± 0.6	16.88 ± 0.77^a^	62 ± 1.0	18.00 ± 1.54^a^
	**150**	58 ± 1.3	22.22 ± 1.54^a^	57 ± 0.2	24.44 ± 0.77^a^	37 ± 1.0	50.00 ± 0.77^b^
	**300**	39 ± 1.0	48.88 ± 0.77^a^	35 ± 0.4	52.88 ± 0.78^a^	26 ± 0.77	65.78 ± 1.54^b^
	**500**	16 ± 0.5	78.22 ± 0.77^a^	12 ± 0.4	83.55 ± 0.76^a^	7 ± 0.4	91.55 ± 1.00^b^

Note: Data are reported as mean ± SD (n = 3). Different superscript letters (a, b) within the same row indicate significant differences according to Fisher’s LSD test (p < 0.05).

Plant toxicity studies show that EC_50_ values—the concentrations causing a 50% reduction in growth—vary widely across plant species and soil types. Reported EC₅₀ values range from 36–536 mg Cu/kg or 22–851 mg Cu/kg, depending on the measured endpoint and soil properties such as pH, organic matter content, and cation-exchange capacity [55]. Based on these data, the concentrations of nanocomposite used in this study (<50 ppm) are considered environmentally safe and suitable for further investigation.

The CuI/Cu^2+^–TiO_2_ nanocomposite exhibited remarkable antifungal activity under visible-light LED irradiation. Evidence from recent studies indicates that Cu-modified TiO₂ materials can release Cu^+^/Cu^2+^ ions that strongly associate with fungal cell walls, leading to membrane destabilisation, leakage of cellular components, and suppression of hyphal growth [56]. Beyond ion-release toxicity, copper species anchored on the nanocomposite surface also contribute to contact-mediated killing, in which copper binds directly to membrane proteins and causes deformation or rupture of the fungal cell envelope. Sensitivity and cellular resistance are enhanced when copper is at the nanoscale [57–58]. After entering the cell, copper ions interfere with crucial metabolic functions by targeting thiol-containing enzymes, disrupting mitochondrial respiration, and disturbing redox balance, ultimately resulting in metabolic failure [59]. In addition, the band-edge positions of CuI/Cu^2^ ⁺ –TiO₂ suggest that visible-light irradiation can promote photocatalytic reactions. Based on established photocatalytic principles, these reactions may generate reactive oxygen species (ROS), such as ●O₂⁻ and ●OH, thereby enhancing oxidative stress against fungal cells [60,61] Fig 11:


$$\begin{array}{c} {\text{Cu}}^{+}\;+\;{\text{O}}_{\text{2}}\;\to \;{\text{Cu}}^{\text{2}+}\;+\;\cdot {\text{O}}_{\text{2}}^{-} \end{array}$$ (10)

$$\begin{array}{c} {\text{Cu}}^{\text{2}+}\;+\text{ Cellular reductants }\to \;{\text{Cu}}^{+} \end{array}$$ (11)

$$\begin{array}{c} {\text{Cu}}^{+}\;+\;{\text{H}}_{\text{2}}{\text{O}}_{\text{2}}\;\to \;{\text{Cu}}^{\text{2}+}\;+\;{\text{OH}}^{-}\;+\;\cdot \text{OH} \end{array}$$ (12)

The TiO₂ framework plays an essential supporting role by stabilising and dispersing copper species, improving surface interaction with fungal cells, and prolonging ion release, resulting in a synergistic antifungal effect that surpasses the performance of either CuI or Cu^2+^ alone [62].

To evaluate the effects of concentration and band gap energy (nanocomposite type) on antifungal efficacy, a two-way ANOVA was performed using the data presented in Table 3 relative to the control treatments. As summarized in Table 4, both factors significantly influenced antifungal efficacy. Concentration exhibited a highly significant effect (F(7,14) = 91.32, p < 0.001), indicating that the antifungal response strongly depended on the applied dosage. Likewise, band gap energy (nanocomposite type) significantly affected antifungal efficacy (F(2,14) = 6.21, p = 0.0117). In both cases, the calculated F-values exceeded the corresponding critical F-values (2.76 and 3.74, respectively), confirming the statistical significance of these factors.

**Table 4:** Two-way ANOVA results showing the effects of concentration and band gap on antifungal efficacy.

ANOVA						
Source of Variation	SS	df	MS	F	P-value	F crit
Rows	18247.51763	7	2606.788	91.32038	1.34E-10	2.764199
Columns	354.5623083	2	177.2812	6.210471	0.011729	3.738892
Error	399.6373583	14	28.54553			
						
Total	19001.7173	23				

Fisher’s Least Significant Difference (LSD) post-hoc test at a 95% confidence level (α = 0.05) was applied to evaluate pairwise differences in antifungal efficacy among 1CuT1, 2CuT2, and 3CuT3. Using Equation (2), with an MSerror value of 28.5455, a sample size of n = 8 concentrations (Table 4), and a critical t-value (tcrit) of 2.145, the calculated LSD value was 5.73%.

The absolute differences between the mean inhibition values at equivalent concentrations were compared with this critical LSD value. At low concentrations (25–100 ppm), most differences among the three nanocomposites were below the LSD threshold, indicating statistically comparable antifungal activities. An exception was observed at 50 ppm, where 3CuT3 exhibited significantly higher inhibition (12.89%) than 1CuT1 (7.11%). A clear divergence in antifungal performance emerged at 150 ppm. At this concentration, 3CuT3 achieved 50.00% inhibition, significantly exceeding the values obtained for 1CuT1 (22.22%) and 2CuT2 (24.44%) (p < 0.05).

This enhanced performance persisted at higher concentrations and reached a maximum at 500 ppm, where 3CuT3 exhibited the highest inhibition efficiency (91.55%), compared with 83.55% for 2CuT2 and 78.22% for 1CuT1. The difference between 1CuT1 and 2CuT2 was not statistically significant (Δ = 5.33% < LSD), whereas the differences between 2CuT2 and 3CuT3 (Δ = 8.00% > LSD) and between 1CuT1 and 3CuT3 (Δ = 13.33% > LSD) were statistically significant. Overall, the inhibition differences between 3CuT3 and the other two nanocomposites exceeded the critical LSD value of 5.73%, demonstrating the superior antifungal performance of 3CuT3. To visually represent these statistical differences, superscript letters (a, b) were used in Table 3 to indicate significant differences among treatments (p < 0.05).

**Fig 12:**
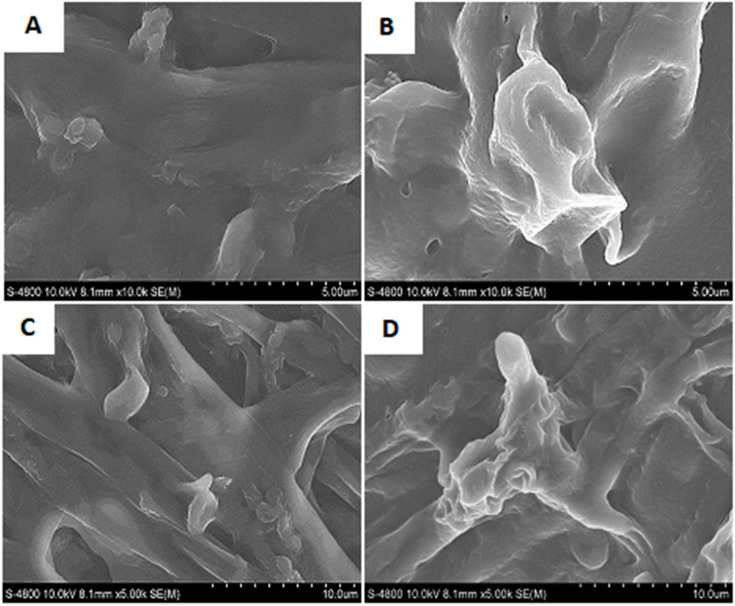
SEM images of M. oryzae grown on control (A, C) and CuI/Cu^2+^–TiO_2_ containing disks (B, D). Scale bars: 5 μm (A, B) and 10 μm (C, D).

As shown in Fig 12, FE-SEM imaging reveals marked morphological damage in *M. oryzae* treated with the CuI/Cu^2+^–TiO₂ nanocomposite. The affected hyphae display clear pore formation, disrupted cell-wall integrity, and pronounced twisting and shrinkage. In contrast, the untreated control exhibits smooth surfaces and normal hyphal growth. Similar structural degradations have been documented in previous studies on the antifungal activity of copper and copper-based formulations against *Colletotrichum gloeosporioides* and *Fusarium kuroshium* using microscopic analysis [63,64]. These observations further support the proposed mechanism underlying the antifungal efficacy of Cu^+^/Cu^2+^ containing systems, such as the CuI/Cu^2+^–TiO_2_ nanocomposite used in this study.

## 4. Conclusion

This study successfully synthesised and characterised a CuI/Cu^2+^–TiO_2_ heterostructured nanocomposite as a sustainable and potent alternative to conventional chemical fungicides. The primary goal was to develop an eco-friendly material capable of suppressing rice blast disease caused by *Magnaporthe oryzae*

Structural and surface characterisation confirmed the successful integration of CuI and Cu^2+^ species with TiO_2_, resulting in the formation of an effective heterojunction. The incorporation of copper species enhanced visible-light utilisation and improved charge separation. The antifungal activity of the CuI/Cu^2+^–TiO_2_ nanocomposite is primarily attributed to the Cu^+^/Cu^2+^ redox cycle, which continuously generates reactive oxygen species (ROS) and releases bioactive copper ions. This synergistic process induces oxidative stress and cellular damage in *M. oryzae*, demonstrating the effectiveness of the nanocomposite as a dual-action antifungal material.

Overall, the CuI/Cu^2+^–TiO_2_ nanocomposite functions as an efficient visible-light-responsive photocatalyst with strong antifungal performance and potential agronomic benefits. Its high efficacy at environmentally relevant concentrations (<50 ppm) highlights its potential as a next-generation nanobiopesticide for sustainable agriculture and long-term food security. Future studies should evaluate its long-term stability, environmental safety, and phytotoxicity under field-relevant conditions. Further investigations are also required to clarify its antifungal mechanisms and assess its effectiveness against a wider range of phytopathogenic fungi.

## Supporting information

S1 File Original EDS spectrum and elemental mapping for Figs 1 and 2. (PDF)

S2 File Raw FE-SEM images for Fig 3. (DOCX)

S3 File Raw data for Fig 4. (XLSX)

S4 File Raw HR-TEM images for Figs 5A and 5B. (DOCX)

S5 File Raw data for Fig 6. (XLSX)

S6 File Raw data for Fig 7. (XLSX)

S7 File Raw data for Fig 8. (XLSX)

S8 File Raw data for Fig 9. (XLSX)

S9 File Raw data for Fig 11. (XLSX)

S10 File Raw FE-SEM images for Fig 12. (DOCX)

S11 File Python code used for band gap calculation. (TXT)
