# Supplementary material for: Photocatalytic performance of CuI/Cu2+ -TiO2 for controlling Magnaporthe oryzae infection in rice plants
Source: PLoS One. 2026 Aug 6;21(8):e0354519. doi: 10.1371/journal.pone.0354519 (PMC13446677; doi:10.1371/journal.pone.0354519)

IMG1(1st)

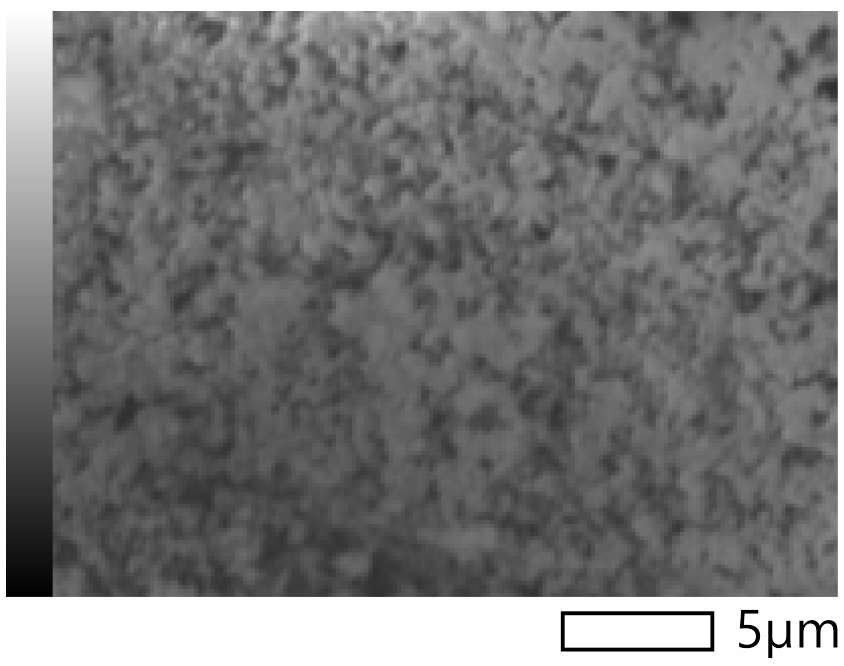

IMG1

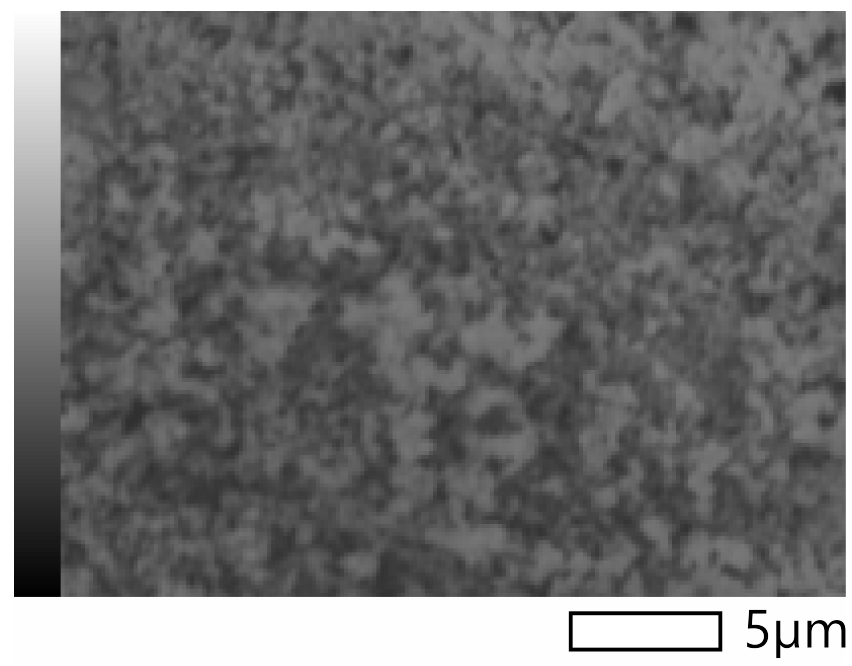

O-K

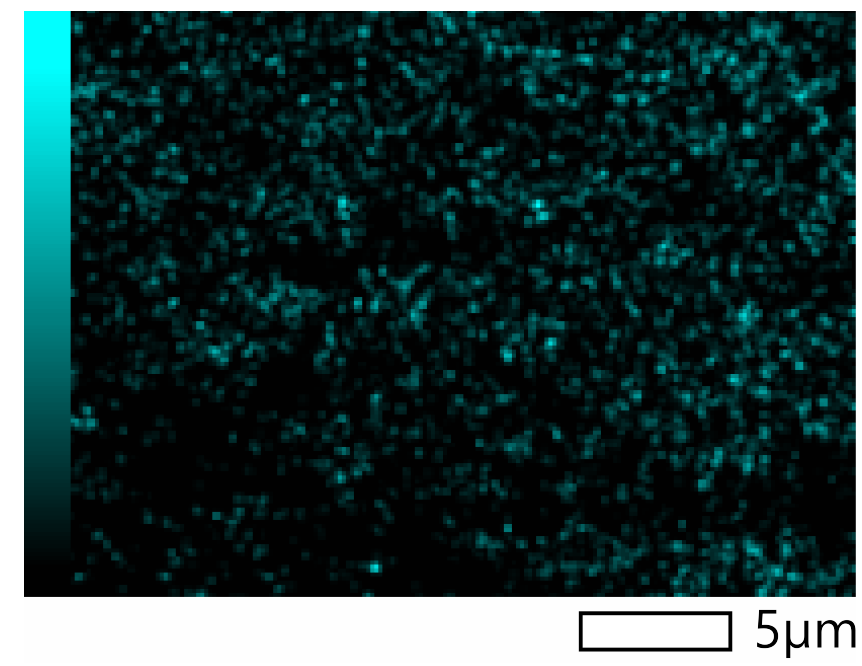

Ti-K

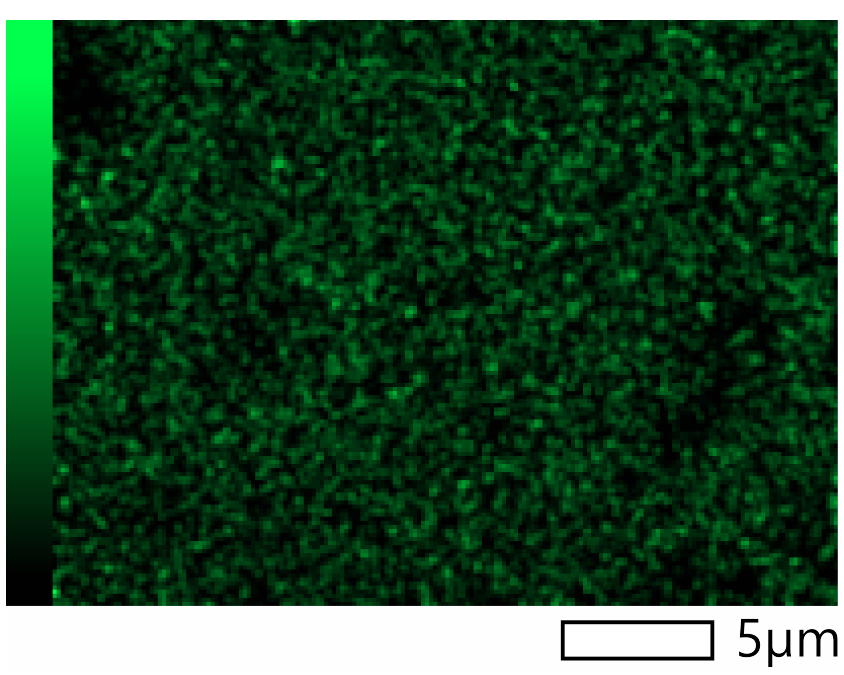

Cu-L

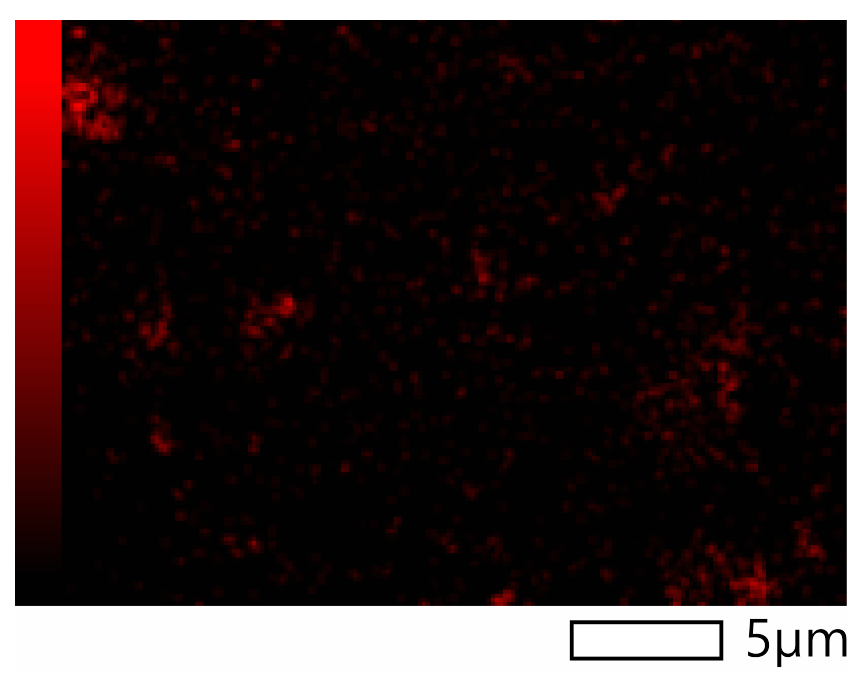

I-L

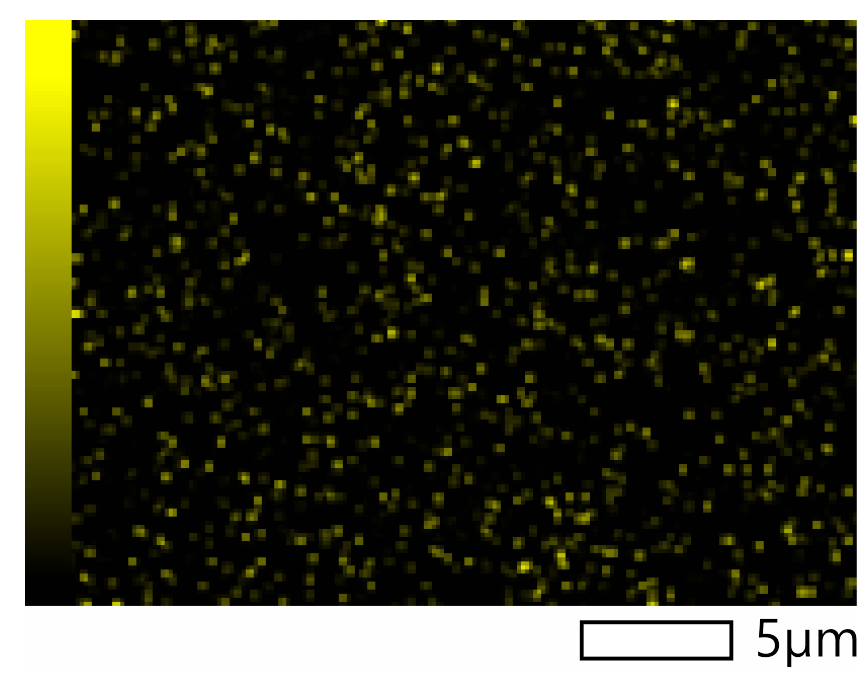

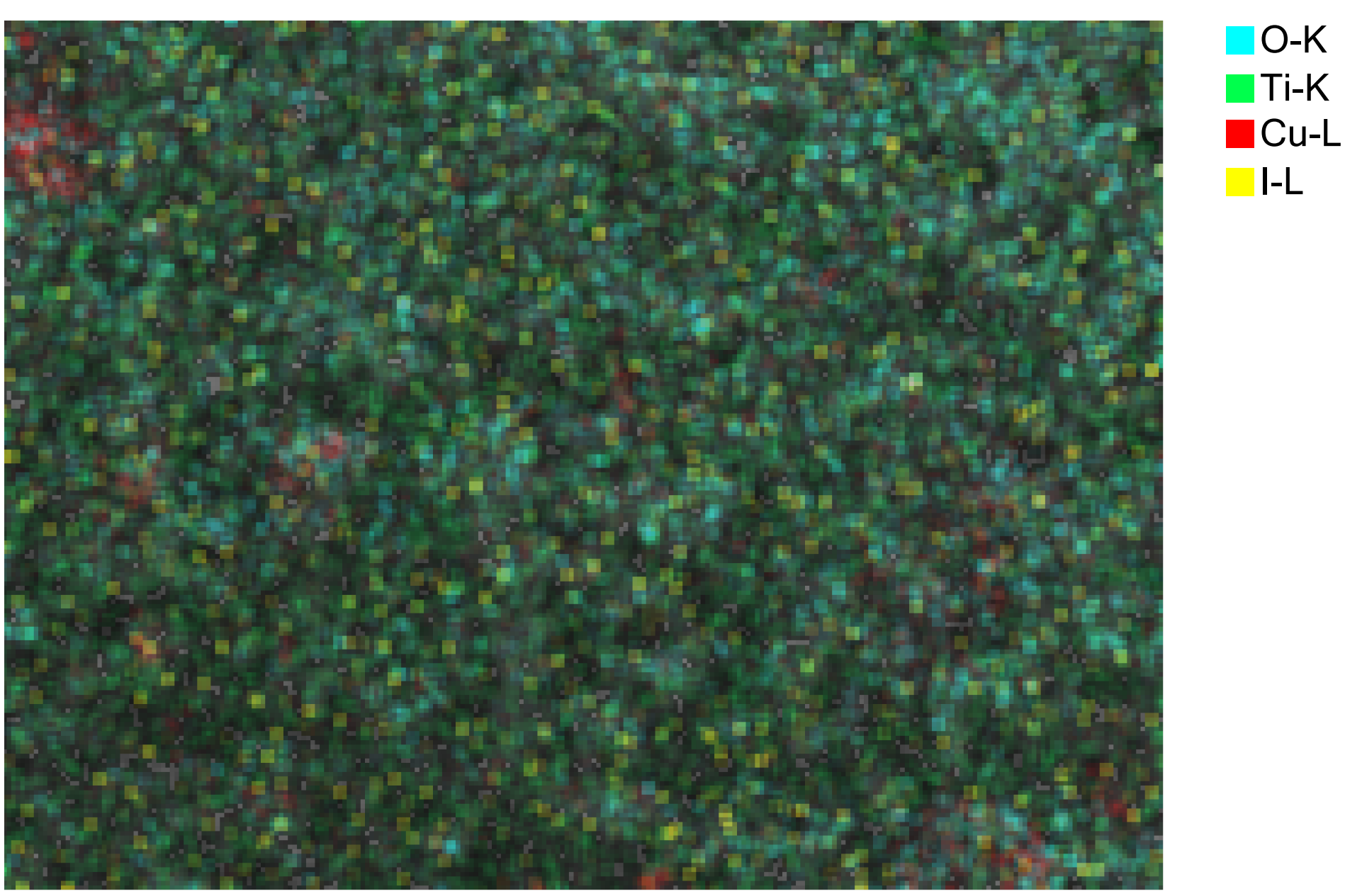

Display name	Standard data	Quantification method	Result Type
Map_009_wholespectrum	Standardless	ZAF	Metal

Element	Line	Mass%	Atom%
O	K	35.91±0.20	62.92±0.36
Ti	K	61.31±0.47	35.88±0.28
Cu	L	2.66±0.09	1.17±0.04
I	L	0.12±0.12	0.03±0.03
Total		100.00	100.00
Map_009_wholespectrum			Fitting ratio 0.0430

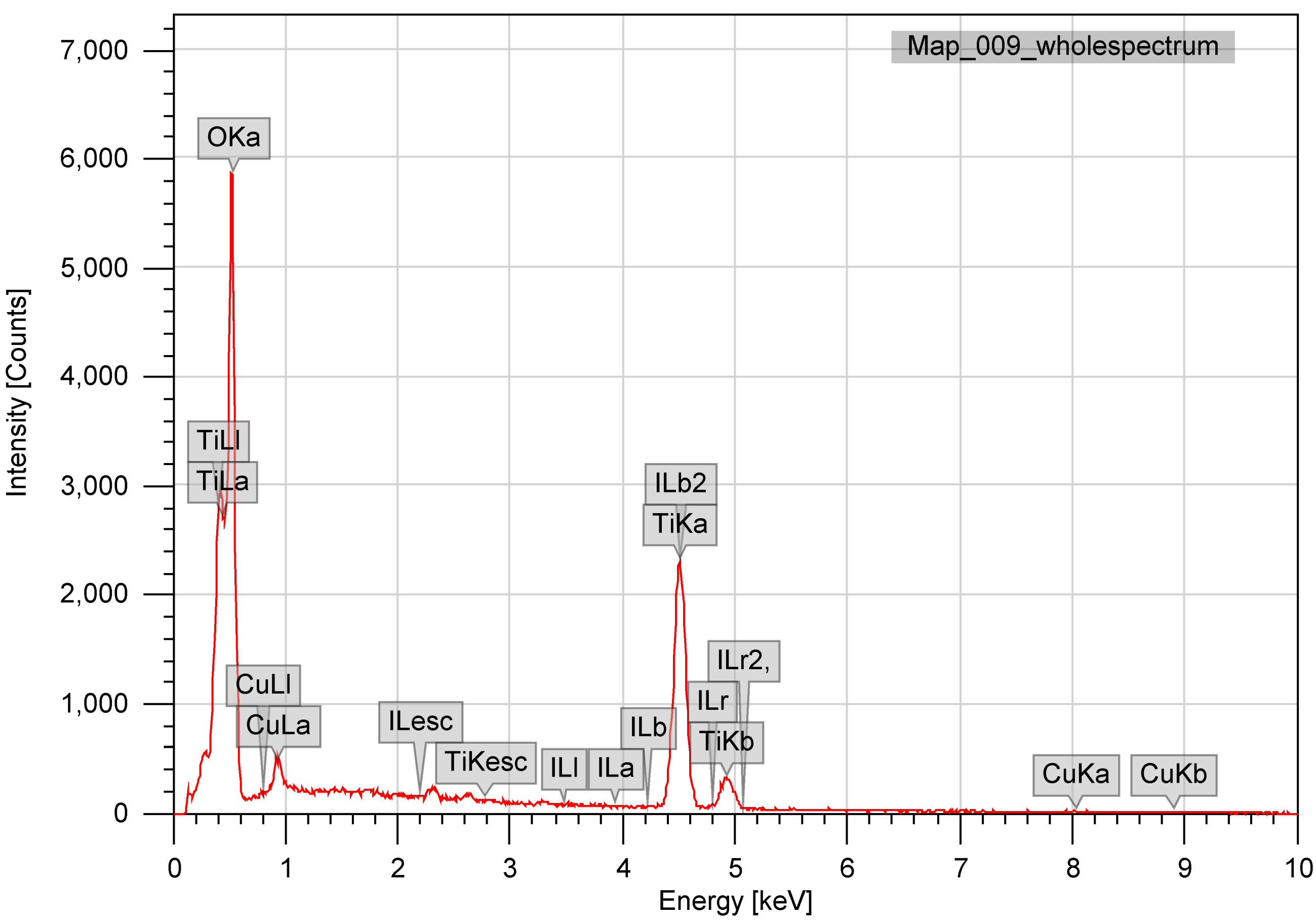

Supplement: Supplementary file 1 [file pone.0354519.s001.pdf]
