# Supplementary material for: Photocatalytic performance of CuI/Cu2+ -TiO2 for controlling Magnaporthe oryzae infection in rice plants
Source: PLoS One. 2026 Aug 6;21(8):e0354519. doi: 10.1371/journal.pone.0354519 (PMC13446677; doi:10.1371/journal.pone.0354519)
Supplement: Supplementary file 2 [file pone.0354519.s002.docx]

**Fig 3A**

**Fig 3B**

**Fig 3C**

**Fig 3D**
